# Lifecourse socioeconomic changes and late-life cognition in a cohort of U.S.-born and U.S. immigrants: findings from the KHANDLE study

**DOI:** 10.1186/s12889-021-10976-6

**Published:** 2021-05-13

**Authors:** Rachel L. Peterson, Kristen M. George, Paola Gilsanz, Elizabeth Rose Mayeda, M. Maria Glymour, Oanh L. Meyer, Dan M. Mungas, Charles DeCarli, Rachel A. Whitmer

**Affiliations:** 1 Biomedical Sciences 1C, University of California Davis School of Medicine, One Shields Ave., Davis, CA 95616 USA; 2 Kaiser Permanente Northern California Division of Research, Oakland, CA USA; 3 University of California Los Angeles School of Public Health, Los Angeles, CA USA; 4 University of California San Francisco School of Medicine, San Francisco, CA USA

**Keywords:** Lifecourse, Socioeconomic status, Cognitive aging, Immigrants

## Abstract

**Background:**

Low socioeconomic status (SES) in early and late life has been associated with lower late-life cognition. Less is known about how changes in SES from childhood to late life are associated with late-life cognition, especially among diverse populations of older adults.

**Methods:**

In a multi-ethnic sample (*n* = 1353) of older adults, we used linear regression to test associations of change in comprehensive measures of SES (financial, cultural, and social domains) from childhood to late life with semantic memory, episodic memory, and executive function. We tested whether the association between SES trajectory and late-life cognition differed by populations who resided in the U.S. during childhood or immigrated to the U.S. as adults.

**Results:**

Participants with low childhood/high late-life financial capital had better semantic memory (β = 0.18; 95% CI: 0.04, 0.32) versus those with low financial capital in both childhood and late life, regardless of childhood residence. We observed a significant interaction in the association of verbal episodic memory and cultural capital by childhood residence (*p* = 0.08). Participants with a foreign childhood residence had higher verbal episodic memory if they had low childhood/high late-life cultural capital (β = 0.32; 95% CI: 0.01, 0.63), but lower verbal episodic memory if they had high childhood/low late-life cultural capital (β = − 0.40; 95% CI: − 0.94, 0.13). Having high lifecourse social capital was associated with better verbal episodic memory scores among those with a U.S. childhood (β = 0.34; 95% CI: 0.14, 0.55), but lower verbal episodic memory among those with a foreign childhood (β = − 0.10; 95% CI: − 0.51, 0.31).

**Conclusions:**

High financial and cultural capital in late life is associated with better cognition, regardless of early childhood SES or childhood residence.

**Supplementary Information:**

The online version contains supplementary material available at 10.1186/s12889-021-10976-6.

## Background

Cognitive impairment and dementia in late life is a growing public health issue with substantial racial/ethnic and socioeconomic disparities [1–3]. Several studies have found higher rates of dementia among immigrant populations in the U.S. and western Europe [4–6]. As the population of older adults in the U.S. diversifies, it is critical to understand the relationships between lifecourse experiences and late-life cognition.

Prior work has demonstrated the importance of early life conditions for dementia incidence [7, 8]. Other studies have observed a positive association between childhood socioeconomic status (SES) and midlife and late-life cognitive outcomes [9–13], while some have found that these relationships are mediated by adult SES [11, 12, 14–16] and/or childhood cognitive abilities [11–13]. Few have considered how changes in SES across the lifecourse are associated with late-life cognitive outcomes. One prior study using the Health and Retirement Survey (15% non-white participants) found that high lifecourse SES predicted higher cognition and slower cognitive decline [17]. The authors further found that education was most strongly associated with baseline cognition while late-life income was most strongly associated with rate of cognitive decline [17]. Another study among an ethnically Mexican cohort residing in the U.S. found that incident cognitive impairment and dementia were highest among those with low SES across the lifecourse, followed by those with low childhood SES and low educational attainment but otherwise high adulthood SES [18]. However, as the older adult population grows more heterogenous, new research is needed to understand how lifecourse SES patterns predict late-life cognitive health in diverse populations. Lifecourse SES may intersect with other experiences and identities, contributing to differing patterns of association. One such intersecting experience may be SES and immigration. This question is particularly poignant for today’s older adults, who experienced a rapidly changing political economy and cultural climate. Changes in immigration policy in 1965 allowed for more arrivals from Latin America and Asia as opposed to Europe, which, coupled with racism and increasing socioeconomic inequality, have been theorized to alter assimilation and socioeconomic opportunities for these groups [19].

Using Bourdieu’s forms of capital [20] as a theoretical framework and insights from prior validation of measuring childhood SES in older adults [21], we constructed comprehensive measures of childhood and late-life SES defined by three domains: financial, cultural, and social capital. This approach to operationalizing SES allows for an explicit examination of the specific experiences and resources embedded in social status, and how these might differentially impact late-life cognition in diverse groups. Financial capital is an economic indicator based on income and/or wealth that is commonly used to operationalize SES. Cultural capital is a reflection of both “material and symbolic profits” that are typically transmitted from one generation to the next and can be operationalized via educational attainment [20]. This conceptualization of education is important in the context of late-life cognition, as it acknowledges the indirect pathway of education on late-life cognitive health through its role in transmitting status and opportunity [22]. Often education is hypothesized to operate in dementia and cognitive aging research via a direct pathway: cognitive stimulation. Social capital is defined as access to real or potential resources available through one’s social connections, and includes benefits that are often intertwined with economic and cultural capital [20, 23]. Operationalization of social capital has been inconsistent, and often differs by discipline [24]. In this analysis, we operationalized social capital as having trusting close relationships that theoretically provide social support and psychological wellbeing [25].

Our study objective was to examine how changes to these three forms of capital from childhood to late adulthood are associated with late-life cognition in a diverse cohort of Asians, Blacks, Latinos, and Whites residing in the U.S. We additionally sought to explore whether these patterns differ based on country of birth and residence during childhood (U.S. vs. non-U.S.). This study adds to the existing literature in several ways. First, it is among very few studies to examine how SES *changes* across the lifecourse may be associated with late-life cognition in a racially/ethnically diverse cohort. Secondly, this study examines how different lifecourse experiences – specifically, being born and raised in the U.S. versus immigrating to the U.S. after childhood – may alter the patterns of association between lifecourse SES and late-life cognition. Finally, we examine a more comprehensive measure of SES, which provides insights into how SES may be associated with late-life cognition.

## Methods

### Sample

We used data from Wave 1 of the Kaiser Healthy Aging and Diverse Life Experiences (KHANDLE) cohort. This cohort consists of 1712 community-dwelling older adults who reside in the San Francisco Bay area and Sacramento valley and are long-term members of Kaiser Permanente Northern California (KPNC). KPNC members demographically reflect residents in the region, though extreme tails of the income distribution are underrepresented [26, 27]. KPNC members were contacted for study recruitment if they were age 65 or older on January 1, 2017; did not have an electronic medical record diagnosis of dementia or another health condition that would impede participation in study interviews (hospice activity in the past 12 months, history of severe chronic obstructive pulmonary disease in the past 6 months, congestive heart failure hospitalizations in the past 6 months, and history of end stage renal disease or dialysis in the past 12 months); and had participated in one or more KPNC Multiphasic Health Checkups (MHC) between 1964 and 1985. MHC were optional medical visits offered as part of routine care to members in San Francisco and Oakland, California that included questionnaires and clinical examination on various modifiable health risk factors.

Of the 80,270 KPNC members contacted, 1115 were excluded for not speaking English or Spanish and 3381 identified as a race/ethnicity other than Asian, Black, Latino and/or White. Lastly, 6262 were excluded due to an electronic medical record diagnosis of dementia or other neurodegenerative disease (*n* = 5115), or presence of health conditions that would impede participation in study interviews (*n* = 1111). Stratified random sampling by race/ethnicity and educational attainment was then used with the goal of recruiting approximately equal proportions of Asian, Black, Latino, and White participants and diversity in educational attainment. Participants were interviewed in their homes or at KPNC clinics from March 2017 to December 2018. The study was approved by the KPNC and University of California Davis Institutional Review Boards and all enrolled participants provided written informed consent.

### Measures

#### Cognition

Our cognitive outcomes of semantic memory, verbal episodic memory, and executive function were derived from the Spanish and English Neuropsychological Assessment Scales (SENAS) [28, 29]. This battery of cognitive tests was developed using item response theory methodology for valid comparisons of cognition and cognitive change across racial/ethnic and linguistically diverse groups. The verbal episodic memory score is derived from a multi-trial word-list-learning test [28, 29]. The semantic memory measure is a composite of highly correlated verbal (object-naming) and nonverbal (picture association) tasks [28, 29]. The executive function composite is constructed from component tasks of category fluency, phonemic (letter) fluency, and working memory (digit-span backward, list sorting) [28, 29]. Details of the administration procedures, development, and psychometric characteristics have been described in detail elsewhere [28, 29]. The SENAS was administered during Wave 1 interviews in either English or Spanish. Each cognitive domain was z-standardized using the Wave 1 sample mean and standard deviation.

#### Socioeconomic trajectories

Childhood and late-life SES were assessed by self-reported measures from KHANDLE Wave 1 that we theoretically mapped onto the constructs of financial, cultural, and social capital [20]. Table 1 provides a crosswalk between the KHANDLE measures and forms of capital. Financial capital was assessed with the following questions: 1) Childhood financial status was considered as low if 1) the participant reported ever having to go hungry due to financial circumstances; or 2) the participant reported their childhood family finances were poor relative to others and that their parents did not own their childhood home. In late life, financial capital is low if the participant meets any of the following criteria: receives supplemental security income or state/county welfare; receives financial assistance from friends or family; has a household income less than $55,000 annually, or is often worried about having enough money to pay for living expenses and has a household income less than $75,000 annually. Although these income cutoffs are relatively high, the values were chosen based on the median household 2018 income in California of $75,000 and the high cost of living in northern California where participants currently reside [30].

**Table 1:** KHANDLE measures used to assess forms of capital in childhood and late adulthood

	Childhood	Late Adulthood
**Low Financial Capital**	Q: “When you were growing up, how often did you skip a meal or go hungry because there was not enough money to buy food?” A: Ever had this experience vs. never	Q: “What was the total income (before taxes) for you and your spouse in the past year?” Q: “Which sources of income do you or your spouse receive?” A: Receives supplemental security income or cash assistance from state/county welfare program or help from family/friends or has household income <US$55 K.
	Q: “Think about your family when you were growing up, from birth to age 16. Would you say your family during that time was pretty well off financially, about average, or poor?” A: Poor vs. well off/about average	
	Q: “During your childhood, did your family mainly: rent, pay a mortgage/own a home, have other living arrangements?” A: Parents rented vs. owned home/other living arrangements	Q: “How often do you worry about not meeting your expenses with your current income?” A: Always/often vs. Never Sometimes AND has household income <US$75 K
**Low Cultural Capital**	Q: “What was the highest educational level of your (mother/father) or the (woman/man) who raised you?” A: One or both parents had ≤8th grade education.	Q: “What is the last grade or highest level in school you completed and got credit for?” A: ≤High School diploma/GED
**Low Social Capital**	Q: “During your childhood, how often was there someone in whom you could talk to, trust and confide?” A: None/a little of the time vs. some/most/all of the time.	Q: “Is there any one special person you know that you feel very close and intimate with -- someone you share confidences and feelings with, someone you feel you can depend on?” A: No vs. Yes

Low childhood cultural capital was defined as having one or both parents with no more than an 8th-grade education while low late-life cultural capital was defined as the participant having a high school diploma/passing the general educational development (GED) test, or less. Although many participants completed their education as young adults, we classified educational attainment as a later-life construct as KPNC records indicate that a portion of KHANDLE participants (about 35% of women and 25% of men) obtained additional education between ages 25 and 65. We varied the cut-offs for low childhood and late-life cultural capital because of generational and historical differences in the level of educational attainment expected and required to attain an average level of social status.

We defined social capital as having someone in whom the participant trusts/trusted that they can confide in and depend on. Low social capital in childhood was defined by participant report of rarely or sometimes having a confidante. In late-life, low social capital was defined as not having one person in whom the participant could trust and depend on.

Using these dichotomized measures, we created four SES trajectories for each form ofcapital: 1) consistently high capital; 2) high childhood and low late-life capital; 3) high late-life and low childhood capital; 4) consistently low capital.

#### Demographics

Participants self-reported their country of birth and, if applicable, the age at which they immigrated to the U.S. We constructed an indicator variable of childhood residence where participants were classified as having a foreign childhood if they were born outside of the U.S. and did not immigrate to the U.S. until they were age 16 or older. Race/ethnicity was self-reported at participant interview, or obtained from the participant’s medical record when missing, and classified as non-Latino White, non-Latino Black, non-Latino Asian, or Latino. Gender and age at KHANDLE Wave 1 were obtained from participant medical records.

### Analysis

Participant demographics and prevalence of low childhood and late-life forms of capital are presented among those who did and did not reside in the U.S. during childhood. We tested associations between the forms of capital in childhood and adulthood using Spearman’s rank correlation with significance at *p* = 0.05. In our sample, 201 (11.4%) were missing one or more measures of childhood capital; of these, 195 were missing both paternal and maternal educational attainment. Forty-nine (2.9%) of the participants were missing one or more measures of late-life capital; 27 were missing information on childhood residence, three were missing information on all three cognitive domains and one was missing race/ethnicity. To correct for missingness in our multivariable analyses we used Multiple Imputation with Chained Equations (MICE) with 5 imputations to account for missingness in all models. For each form of capital, linear regression was used to examine the association between socioeconomic trajectory and late-life cognition, adjusting for age at KHANDLE Wave 1, gender, race/ethnicity, and childhood residence. In separate analyses, we evaluated whether childhood residence modified the association between socioeconomic trajectory and late-life cognition and we estimated the association between socioeconomic trajectory and late-life cognition in models stratified by childhood residence. To assess the impact of unmeasured confounding in our primary analyses, we calculated E-values following the method set forth by VanderWeele and Ding (2017) [31]. E-values are calculated as risk ratios and reflect the robustness of an association to potential unmeasured or uncontrolled confounding. Higher E-values increasing confidence that an association exists [31, 32].. All analyses were performed in Stata 14.2 (StataCorp, College Station, TX).

## Results

### Summary statistics

About 28% of participants were born and resided outside of the U.S. until at least age 16. Among those with a childhood residence outside of the U.S., 51% were Asian, 31% were Latino, 17% were White, and 1% were Black. By contrast, 35% of participants with a U.S. childhood residence were White, 28% were Black, 19% were Asian, and 18% were Latino (Table 2). Although there was no difference by childhood residence in prevalence of low financial capital in childhood or late life, we observed differences in some of the individual measures of financial capital. Those residing outside of the U.S. were more likely to report experiencing childhood hunger (17% vs. 12%), while those who resided in the U.S. as children were more likely to report being poor in childhood relative to others they knew (35% vs. 27%), and more likely to report late-life financial worries (22% vs. 15%). Those who resided outside of the U.S. had lower childhood and late-life cultural capital than those who resided in the U.S. as children. Additionally, participants residing outside of the U.S. as children were more likely to have low late-life social capital (14% vs. 10%), though there was no difference by residence in low childhood social capital.

**Table 2:** Demographic characteristics of KHANDLE participants by childhood residence

	US Childhood (***n*** = 1058)	Foreign Childhood (***n*** = 295)
Age (mean, SD)	75.1 (6.5)	76.3 (6.2)
Female (n, %)	636 (60.1)	170 (57.6)
**Race/Ethnicity**		
Asian	202 (19.1)	151 (51.2)
Black	299 (28.3)	3 (1.0)
Latinx	191 (18.1)	90 (30.5)
White	366 (34.6)	51 (17.3)
**Low Childhood Financial Capital**	**292 (24.6)**	**73 (24.8)**
Ever go hungry due to finances	123 (11.7)	50 (17.1)
Parents did not own childhood home	405 (38.4)	112 (38.4)
Family was poor relative to others	338 (34.5)	76 (27.0)
**Low Childhood Cultural Capital**		
Father and/or mother earned ≤8th-grade education	388 (36.7)	154 (52.2)
**Low Childhood Social Capital**		
Never/sometimes had someone trust and confide	401 (37.9)	111 (37.6)
**Low Late-Life Financial Capital**	**468 (39.5)**	**107 (36.3)**
vParticipant receives SSI, state welfare, support from friends/family or earns < 55 K annually	348 (33.0)	102 (35.1)
Always/often worries about having enough to cover living expenses AND earns < 75 K annually	208 (21.9)	39 (14.9)
**Low Late-Life Cultural Capital**		
Education ≤High School/General Education Diploma	125 (11.8)	57 (19.3)
**Low Late-Life Social Capital**		
Does not have someone to confide in or can depend on	102 (9.6)	42 (14.2)
**Financial Capital Trajectory**		
Consistently High	501 (47.4)	146 (49.5)
Low Childhood, High Adult	145 (13.7)	42 (14.2)
High Childhood, Low Adult	298 (28.2)	76 (25.8)
Consistently Low	114 (10.8)	31 (10.5)
**Cultural Capital Trajectory**		
Consistently High	615 (58.1)	127 (43.1)
Low Childhood, High Adult	318 (30.1)	111 (37.6)
High Childhood, Low Adult	55 (5.2)	14 (4.8)
Consistently Low	70 (6.6)	43 (14.6)
**Social Capital Trajectory**		
Consistently High	615 (58.1)	165 (55.9)
Low Childhood, High Adult	341 (32.2)	88 (29.8)
High Childhood, Low Adult	42 (4.0)	19 (6.4)
Consistently Low	60 (5.7)	23 (7.8)

We observed weak correlations between forms of capital with only four pairwise combinations correlating above 0.10: childhood and later-life cultural capital (0.18); childhood cultural capital and childhood financial capital (0.18); late-life cultural capital and later-life financial capital (0.15); and childhood and late-life social capital (0.14). In examining SES trajectories, we noted differences by childhood residence in cultural capital trajectories where a higher proportion of participants who resided in the U.S. during childhood had high lifecourse cultural capital (58% vs. 43%) while a higher proportion of participants who resided outside of the U.S. in childhood had low lifecourse cultural capital (15% vs. 7%). We observed limited differences in trajectories of financial or social capital by childhood residence.

### Financial capital and cognitive function

**Fig. 1:**
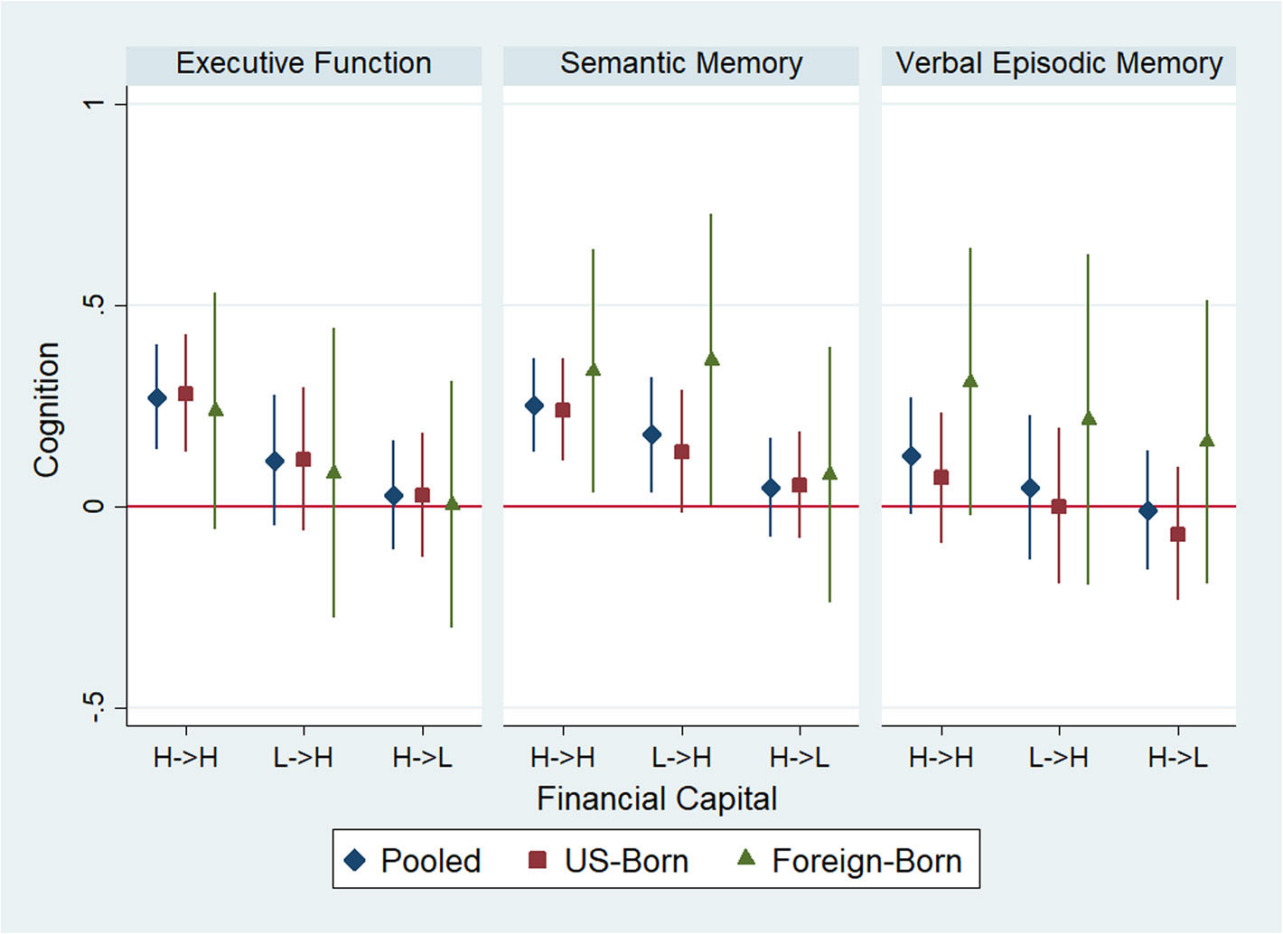
Linear regression with MICE point estimates and 95% confidence intervals for the association of financial capital trajectory and z-standardized late-life cognition, pooled and stratified by childhood residence. H- > H=High Lifecourse Capital; L- > H = Low Childhood, High Late-Life Capital; H- > L = High Childhood, Low Late-Life Capital. Reference is Low Lifecourse Capital

In pooled linear regression models using low lifecourse financial capital as the reference, having high lifecourse financial capital was associated with better executive function (β = 0.28; 95% CI: 0.15, 0.40) and semantic memory (β = 0.25; 95% CI: 0.13, 0.37). The association with verbal episodic memory was about half as large (β = 0.12; 95% CI: − 0.02, 0.26). Those with low childhood/high late-life financial capital also had higher semantic memory (β = 0.18; 95% CI: 0.04, 0.32), (Fig. 1). In stratified analyses, high lifecourse financial capital was associated with better executive function and semantic memory among both participants with a foreign childhood (executive function: β = 0.21; 95% CI: − 0.07, 0.50; semantic memory: β = 0.33; 95% CI: 0.02, 0.63) and a U.S. childhood (executive function: β = 0.29; 95% CI: 0.15, 0.44; semantic memory: β = 0.24; 95% CI: 0.12, 0.37). There was no evidence that place of childhood residence modified the association of lifecourse financial capital with any cognitive domain. In sensitivity analyses we calculated E-values and 95% confidence intervals closest to the null for associations of high lifecourse (versus low lifecourse) financial capital with executive function, semantic memory, and verbal episodic memory of 1.88 (1.52), 1.82 (1.51), and 1.11 (1.00), respectively (Supplemental Table 1). For associations of low childhood and high adult (versus low lifecourse) financial capital and executive function, semantic memory, and verbal episodic memory, we calculated E-values of 1.43 (1.00), 1.61 (1.18), and 1.49 (1.00), respectively. These risk ratios indicate that any unmeasured confounder would need to be between about 1.1 and 1.9 times greater than observed associations to explain away these findings. For comparison, we calculated the E-values for age, if it were an unmeasured confounder in associations of financial capital and executive function, semantic memory, and verbal episodic memory of 1.27 (1.25), 1.26 (1.24), and 1.28 (1.26), respectively.

### Cultural capital and cognitive function

**Fig. 2:**
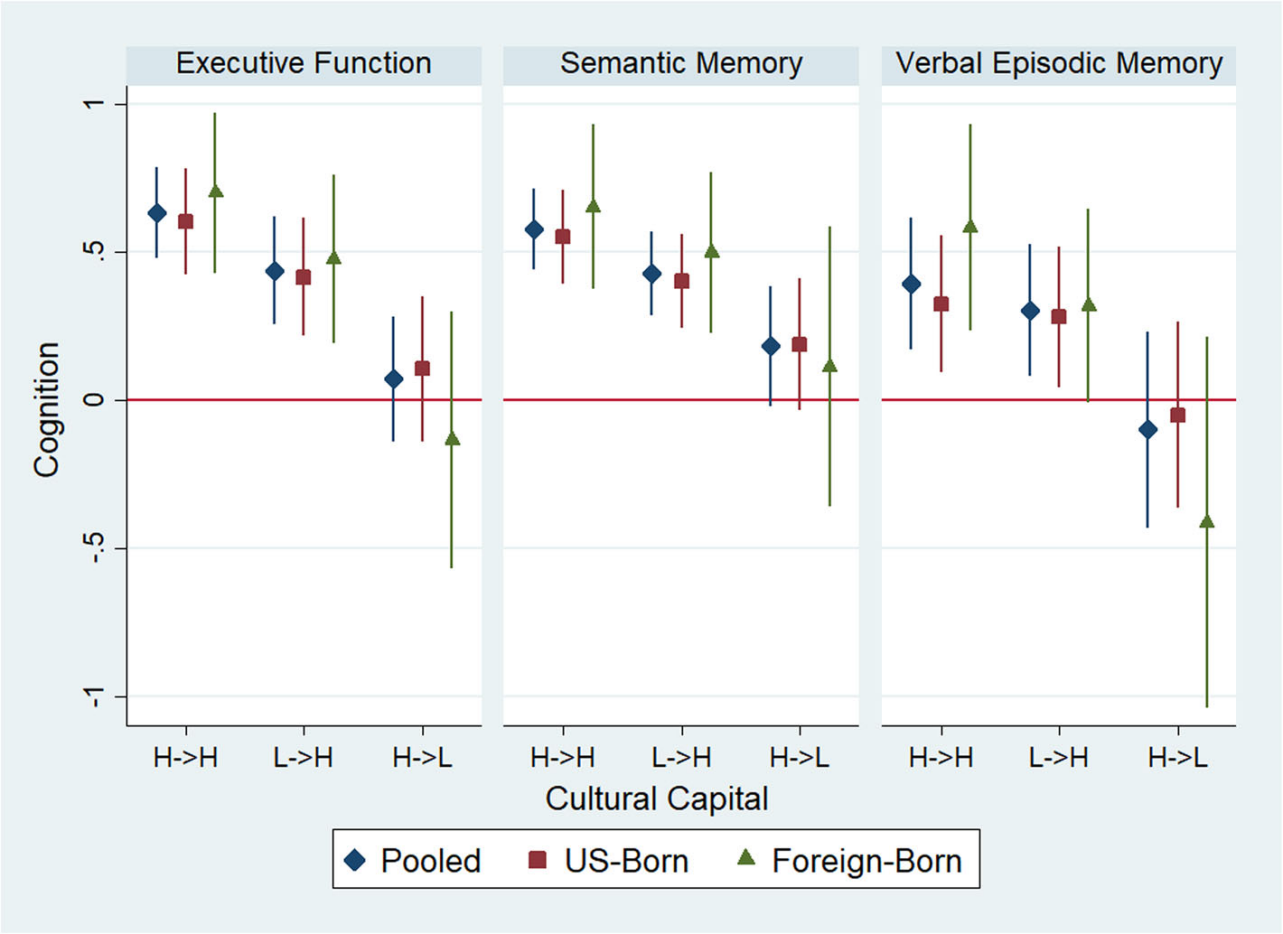
Linear regression with MICE point estimates and 95% confidence intervals for the association of cultural capital trajectory and z-standardized late-life cognition, pooled and stratified by childhood residence. H- > H=High Lifecourse Capital; L- > H = Low Childhood, High Late-Life Capital; H- > L = High Childhood, Low Late-Life Capital. Reference is Low Lifecourse Capital

In pooled analyses using low lifecourse cultural capital as the reference, those with high lifecourse cultural capital and low childhood/high late-life cultural capital were associated with significantly better cognition across all cognitive domains (Fig. 2). We observed a significant interaction in the association of cultural capital with verbal episodic memory (*p* = 0.08). In stratified analyses, having high lifecourse cultural capital compared to low lifecourse cultural capital produced a higher estimated verbal episodic memory score among those with a foreign childhood (β = 0.56; 95% CI: 0.26, 0.86) than those with a U.S. childhood (β = 0.35; 95% CI: 0.11, 0.58). Having low childhood/high late-life cultural capital was associated with better verbal episodic memory among both those with a foreign childhood (β = 0.32; 95% CI: 0.01, 0.63) and a U.S. childhood (β = 0.30; 95% CI: 0.06, 0.53). Having high childhood/low late-life cultural capital was associated with a substantially lower verbal episodic memory score among those with a foreign childhood (β = − 0.40; 95% CI: − 0.94, 0.13), but not a U.S. childhood (β = − 0.03; 95% CI: − 0.33, 0.28).

In sensitivity analyses, we calculated E-values and lower 95% confidence intervals in associations of high lifecourse cultural capital (versus low lifecourse capital) and executive function, semantic memory, and verbal episodic memory, of 2.93 (2.48), 2.74 (2.34), and 2.23 (1.81), respectively. E-values for associations between low childhood and high adult cultural capital (versus low lifecourse capital) with executive function, semantic memory, and verbal episodic memory were 2.33 (1.92), 2.30 (1.93), and 1.95 (1.49), respectively (Supplemental Table 1).

### Social capital and cognitive function

**Fig. 3:**
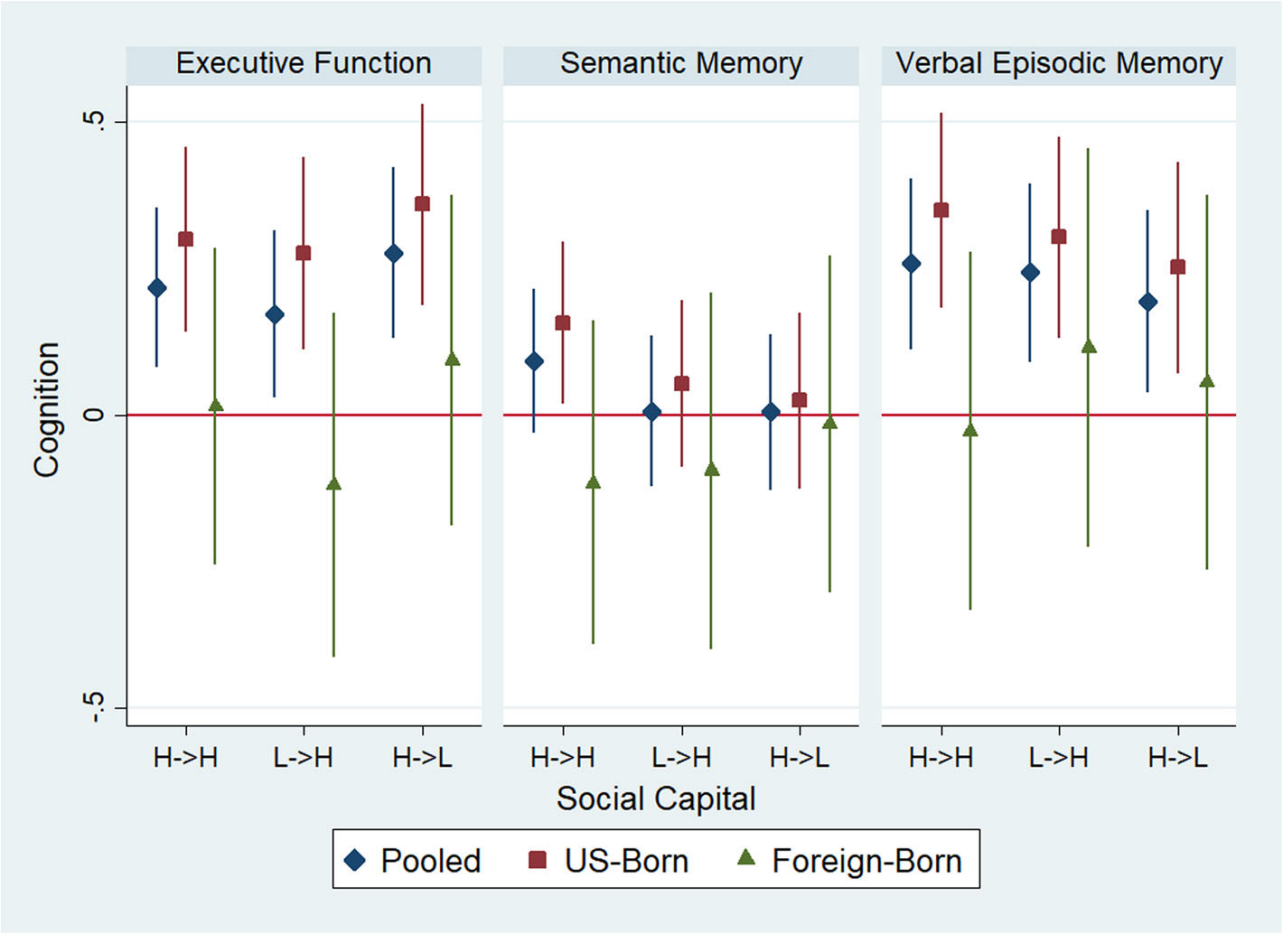
Linear regression with MICE point estimates and 95% confidence intervals for the association of social capital trajectory and z-standardized late-life cognition, pooled and stratified by childhood residence. H- > H=High Lifecourse Capital; L- > H = Low Childhood, High Late-Life Capital; H- > L = High Childhood, Low Late-Life Capital. Reference is Low Lifecourse Capital

Compared with participants who had low lifecourse social capital, we found significantly higher executive function and verbal episodic memory among those with high lifecourse social capital (executive function β = 0.37; 95% CI: 0.20, 0.54; verbal episodic memory β = 0.24; 95% CI: 0.06, 0.42), low childhood/high late-life social capital (executive function β = 0.31; 95% CI: 0.13, 0.48; verbal episodic memory β = 0.20 (0.01, 0.39), and higher executive function among those with high childhood/low late-life social capital (β = 0.41; 95% CI: 0.18, 0.65) (Fig. 3). Pooled effects for differences by lifecourse trajectory in the association of social capital with semantic memory were null. We did not observe significant interactions by childhood residence for any cognitive domain. In stratified models, we observed better executive function and verbal episodic memory scores among those with a U.S.-based childhood who reported high social capital at any point in the lifecourse. Among those with a foreign childhood residence, the effects of high social capital at any point in the lifecourse were associated with better executive function scores but had negative or null associations with semantic memory and verbal episodic memory.

In sensitivity analyses, we calculated E-values and lower-bound 95% confidence intervals in associations of high lifecourse (versus low lifecourse) social capital with executive function, semantic memory, and verbal episodic memory of 2.14 (1.69), 1.29 (1.00), and 1.77 (1.26), respectively. In associations of high adult, low child (versus low lifecourse) social capital with executive function, semantic memory, and verbal episodic memory, we calculated E-values of 1.96 (1.49), 1.13 (1.00), and 1.67 (1.08), respectively (Supplemental Table 1).

## Discussion

In this multi-ethnic sample of older adults, individuals with high adult SES averaged better cognition. Although individuals who had advantaged SES in both childhood and late life generally had the best cognitive outcomes, advantaged childhoods generally did not predict better cognitive outcomes unless the socioeconomic advantage was maintained into adulthood. Similar patterns were observed for financial security and education, although the magnitudes of association between educational advantage and cognitive domains were generally larger than for financial security. We observed some differences in the associations with social capital by childhood residence. Specifically, having high social capital at any point in the lifecourse was associated with better verbal episodic memory among those with a U.S. childhood, but not a foreign childhood. Overall, these findings suggest that SES in late life may be for a better predictor of late-life cognition than childhood SES, regardless of childhood residence.

Our findings are consistent with prior studies of SES that found those with higher late-life SES had better cognition [33–35] and slower cognitive decline [17]. Some prior studies also found that upward mobility was associated with better cognitive outcomes compared to downward mobility or consistently low lifecourse SES [17, 33]. Our study further supports prior findings that the strongest associations for cognitive level are for education compared to financial resources [17, 34]. The observed benefits of education may operate through its direct effect on cognitive stimulation or through selection of individuals into receiving more education. However, our findings for the benefits of high late-life financial capital for all participants and high social capital among participants with a U.S. childhood could also suggest the cognitive benefits of access to material and psychosocial resources in late life. Overall, these findings suggest that policies that support financial opportunities in midlife and late life may be a key approach to reducing the risk of cognitive impairment in late life. This idea is further supported by a natural experiment that found inflation adjustments that increased Social Security income in the 1970s were predictive of better cognitive function and slower cognitive decline [36]. Increasing educational opportunities in midlife and late life could also play an important role in late-life cognition.

Our study adds to this literature by demonstrating that upward mobility in financial and cultural capital is predictive of better late-life cognition in heterogenous populations, while the cognitive penalty of a downward trajectory in cultural capital is associated with worse cognition in participants with a foreign childhood but is null among those with a U.S. childhood.

Our findings for the impact of upward mobility in cultural and financial capital among populations with foreign childhoods are particularly salient in today’s political-economic climate, where many non-White immigrants have experienced a segmented assimilation pattern that limits opportunities for upward mobility and the social and health benefits that correspond with it [37]. These experiences are distinct from those of European immigrants of the early twentieth century and the participants in this study, the majority of whom emigrated from Spanish speaking and Asian countries by 1970, and suggest that limitations to socioeconomic mobility among immigrants today may hinder public health efforts to reduce the risk of cognitive impairment and dementia in an increasingly heterogenous aging population.

Several limitations should be noted. This study relied on cross-sectional data to assess late-life cognition without the ability to control for cognition earlier in life or other potentially time-varying confounders of lifecourse SES. Our findings may be biased by variation in cognition established early in the lifecourse rather than changes resulting from lifecourse SES trajectories [38, 39]. We cannot fully account for the duration of “exposure” for each of these late-life SES indicators as they were assessed in late life but may have been established in young adulthood. Furthermore, early life SES indicators are based on self-reported data and subject to recall bias. Additionally, due to power restrictions this study combined diverse race/ethnic groups into U.S. versus foreign childhood residence. In this study, about half of the foreign childhood group is classified as Asian and are from linguistically and culturally diverse countries. Those classified as Latino in this study predominately emigrated from Mexico. This heterogeneity in the foreign childhood group makes it difficult to disentangle to what extent differences are due to childhood residence versus experiences based on racial/ethnic identity. Finally, participants of this study are long-term members of KPNC and their access to healthcare may not reflect the experience of the broader population.

Nonetheless, this study had several strengths that contribute to our understanding of how SES trajectories may predict late-life cognition. This study explicitly examined how different domains of SES may intersect with other forms of identity – nativity and childhood residence – that shape early and late-life social experiences [40]. Additionally, no prior studies of SES trajectories and late-life cognitive outcomes have, to our knowledge, included a measure of SES equivalent to social capital in this study. This measure is an important component of SES as prior research has suggested that SES may influence health through a psychosocial stress response based on one’s relative position in a social hierarchy [41, 42]. Social capital, as operationalized here, may play an important role in how lifecourse SES influences late-life health outcomes. That we only observed associations between social capital and late-life cognition among those with a U.S. childhood may reflect differences in how social capital is experienced in immigrant populations. Finally, that we observed cognitive benefits of higher late-life SES compared to those with low lifecourse SES in a sample of participants who are decades-long members of KPNC suggests the benefits of higher SES on health go beyond access to quality healthcare.

## Conclusions

Our study found evidence that late-life SES is more strongly associated with late-life cognition than childhood SES. This finding provides optimistic insights for promoting healthy cognitive aging. Public health programs and policies that help to improve SES and increase upward mobility in midlife and late life may help to reduce poor cognitive outcomes among diverse older adults.

## Supplementary Information

**Additional file 1: Supplemental Table 1.** E-values and 95% confidence intervals closest to the null for associations of high lifecourse (versus low lifecourse) financial capital with executive function, semantic memory, and verbal episodic memory. These risk ratios indicate the minimum strength of association that unmeasured confounders would need to have to explain away the primary findings.

## Data Availability

The datasets used during the current study are available from the corresponding author on reasonable request.

## Abbreviations

KHANDLE: Kaiser Healthy Aging and Diverse Life Experiences study
KPNC: Kaiser Permanente Northern California
MHC: Multiphasic Health Checkups
MICE: Multiple Imputation using Chained Equations
SENAS: Spanish and English Neuropsychological Assessment Scale
SES: Socioeconomic Status
