# Supplementary material for: Lifecourse socioeconomic changes and late-life cognition in a cohort of U.S.-born and U.S. immigrants: findings from the KHANDLE study
Source: BMC Public Health. 2021 May 13;21:920. doi: 10.1186/s12889-021-10976-6 (PMC8120825; doi:10.1186/s12889-021-10976-6)
Supplement: Supplementary file 1 — Additional file 1: Supplemental Table 1. E-values and 95% confidence intervals closest to the null for associations of high lifecourse (versus low lifecourse) financial capital with executive function, semantic memory, and verbal episodic memory. These risk ratios indicate the minimum strength of association that unmeasured confounders would need to have to explain away the primary findings. [file 12889_2021_10976_MOESM1_ESM.docx]

**Supplemental Table 1.** E-values and 95% confidence intervals closest to the null for associations of high lifecourse (versus low lifecourse) financial capital with executive function, semantic memory, and verbal episodic memory. These risk ratios indicate the minimum strength of association that unmeasured confounders would need to have to explain away the primary findings.

|  | **Executive Function** | **Semantic Memory** | **Verbal Episodic Memory** |
| --- | --- | --- | --- |
|  | **E-value (lower 95% CI)** | **E-value (lower 95% CI)** | **E-value (lower 95% CI)** |
| **Financial Capital** |  |  |  |
| Consistently Low | Reference | Reference | Reference |
| Low Childhood, High Adult | 1.43 (1.00) | 1.61 (1.18) | 1.49 (1.00) |
| High Childhood, Low Adult | 1.20 (1.00) | 1.26 (1.00) | 1.23 (1.00) |
| Consistently High | 1.88 (1.53) | 1.82 (1.51) | 1.11 (1.00) |
| *Age (E-value if omitted from model)* | *1.27 (1.25)* | *1.26 (1.24)* | *1.28 (1.26)* |
| **Cultural Capital** |  |  |  |
| Consistently Low | Reference | Reference | Reference |
| Low Childhood, High Adult | 2.33 (1.92) | 2.30 (1.93) | 1.95 (1.49) |
| High Childhood, Low Adult | 1.39 (1.00) | 1.58 (1.00) | 1.36 (1.00) |
| Consistently High | 2.93 (2.48) | 2.74 (2.34) | 2.23 (1.81) |
| *Age (E-value if omitted from model)* | *1.26 (1.24)* | *1.25 (1.23)* | *1.27 (1.25)* |
| **Social Capital** |  |  |  |
| Consistently Low | Reference | Reference | Reference |
| Low Childhood, High Adult | 1.96 (1.49) | 1.13 (1.00) | 1.67 (1.08) |
| High Childhood, Low Adult | 2.24 (1.59) | 1.29 (1.00) | 1.57 (1.00) |
| Consistently High | 2.14 (1.69) | 1.29 (1.00) | 1.77 (1.26) |
| *Age (E-value if omitted from model)* | *1.27 (1.26)* | *1.26 (1.24)* | *1.28 (1.26)* |
